# Bevacizumab as a treatment for hereditary hemorrhagic telangiectasia in children: a case report

**Published:** 2017-06-30

**Authors:** Fabio E Ospina, Alex Echeverri, Iván Posso-Osorio, Lina Jaimes, Jaiber Gutierrez, Gabriel J Tobón

**Affiliations:** 1 Instituto de Investigaciones Clínicas, Fundación Valle del Lili, Cali, Colombia.; 2 Grupo de Investigación en Reumatología, Autoinmunidad y Medicina Traslacional (GIRAT) Departamento de Reumatologia, Fundación Valle del Lili, Cali, Colombia.; 3 Facultad Ciencias de la SaludUniversidad Icesi Cali, Colombia; 4 Departamento de Pediatria, Fundación Valle del Lili, Cali, Colombia.

**Keywords:** Telangiectasia Hereditary Hemorrhagic, Bevacizumab, Osler-Rendu-Weber Disease, Pediatrics

## Abstract

**Case description:**

Five-year-old female patient with hereditary hemorrhagic telangiectasia.

**Clinical Findings:**

Deterioration of cardiopulmonary function with higher oxygen requirements secondary to pulmonary arteriovenous shunts, epistaxis.

**Treatment and Outcome:**

The patient was treated with the monoclonal antibody bevacizumab, which inhibits the vascular endothelial growth factor, with good clinical outcome.

**Clinical Relevance:**

Hereditary hemorrhagic telangiectasia is an autosomal dominant disorder characterized by arteriovenous malformations in different organs, making its clinical presentations varied. Systemic therapeutic options for a generalized disease are limited. The monoclonal antibody bevacizumab, seems to be a good option in this disorder. Although reported as successful in adult population, its use in pediatric population has not yet been reported. Here we report the use of bevacizumab in a 5-year-old female patient with hereditary hemorrhagic telangiectasia, showing clinical benefits and good outcome.

## Resumen

**Descripción del caso:**

Paciente de cinco años de sexo femenino con telangiectasia hemorrágica hereditaria.

**Hallazgos Clínicos:**

Deterioro de la función cardiopulmonar con mayores requerimientos de oxígeno secundario a shunt pulmonar arteriovenoso, epistaxis.

**Tratamiento y resultado:**

La paciente fue tratada con el anticuerpo monoclonal bevacizumab, que inhibe el factor de crecimiento endotelial vascular, con buen resultado clínico.

**Relevancia clínica:**

La telangiectasia hemorrágica hereditaria es un trastorno autosómico dominante caracterizado por malformaciones arteriovenosas en diferentes órganos, lo que hace que sus presentaciones clínicas varíen. Las opciones terapéuticas sistémicas para la enfermedad generalizada son limitadas. El anticuerpo monoclonal bevacizumab, parece ser una buena opción en este trastorno. Aunque se ha reportado como exitoso en la población adulta, su uso en población pediátrica aún no ha sido reportado. Aquí se informa el uso de bevacizumab en una paciente de 5 años de edad con telangiectasia hemorrágica hereditaria, mostrando beneficios clínicos y buen resultado.

## Introduction 

Hereditary hemorrhagic telangiectasia (HHT) or Osler-Weber-Rendu syndrome is an autosomal-dominant multisystemic vascular disorder, characterized by mucocutaneous telangiectases and arteriovenous malformations (AVMs) which predisposes to shunts formation and bleeding. Its prevalence is close to 1: 5,000-8,000 ^1^. In most cases, it is associated to mutations in the genes encoding the receptors in the transforming growth factor-β/bone morphogenetic protein (TGF-β/BMP) superfamily. The identified mutations cause impaired endothelial behavior, angiogenesis and/or vascular remodeling ^2^ ^,^ ^3^. Mutations in two different genes are responsible for two subtypes: HHT1 (Eng mutation, mostly pulmonary and cerebral AVMs) and HHT2 (Acvrl1 mutation, mainly hepatic AVMs) ^1^ ^-^ ^3^. 

The number and location of lesions vary, even within the same family. Most telangiectases are in the oral, nasal and gastrointestinal mucosa ^4^; and AVMs may involve lungs ^5^, liver ^6^ and the central nervous system ^7^. Epistaxis is usually, not always, the earliest sign ^1^ ^,^ ^2^. Onset can also be represented by brain abscess, intracranial hemorrhage or pulmonary symptoms ^8^. In pediatric patients with HHT the most common manifestations are epistaxis and mucocutaneous involvement. Other affected organs are lungs and brain, with presence of AVMs even in asymptomatic patients. Large AVMs are frequently associated with complications in childhood ^8^ ^,^ ^9^. Liver involvement can lead to liver failure and high-output cardiac failure; portal hypertension and ischemic biliary disease ^10^. As no effective systemic therapy is currently available, only local treatments are employed, according to the involved organ. By instance, in the treatment of epistaxis several options such as septodermoplasty, surgical artery ligation and laser, among others, are effective for local control. To treat the gastrointestinal bleeding, the use of laser or heat probe have shown short-term benefit ^4^,^11^. The embolization of central nervous system or lungs AVMs has shown to be a good option ^12^. Asymptomatic patients with hepatic AVMs are usually not treated ^10^, ^11^. On the other hand, systemic therapy includes estrogen ^11^, tamoxifen for recurrent bleeding but with restrictions due to their known teratogenic effect ^13^; and tranexamic acid which has shown promising results but with a potential prothrombotic effect ^14^.

Bevacizumab, a recombinant humanized monoclonal antibody that inhibits the vascular endothelial growth factor (VEGF), binding to its receptors on the surface of the endothelial cells, inhibits proliferation of endothelial cells, endothelial growth and cause regression of existing vessels increasing endothelial cell death ^15^. The use of intravenous bevacizumab at doses of 5-10 mg/kg every three weeks in HHT demonstrated decreased episodes of epistaxis and improvement of the high cardiac output secondary to AVMs ^16^, and averts the need of liver transplantation after 6 months of treatment in adult patients with hepatic compromise on HHT ^17^. In this article, we report the case of a pediatric patient with multiple AVMs secondary to HHT, treated successfully with bevacizumab for three months (6 cycles).

## Case report

A 5-year-old female patient was referred to our institution for liver failure and multiple pulmonary arteriovenous fistulas. The mother smoked during the first trimester of pregnancy and congenital cytomegalovirus (CMV) was diagnosed. The child's delivery was at 38 weeks without complications, weight 2,500 g, length 42 cm. Two months after birth the patient developed CMV bronchiolitis obliterans and pneumonia (viral load >120,000 copies), impaired respiratory function and alveolar hemorrhage. She required invasive mechanical ventilation and antiviral therapy. At that time, an abnormal liver function was associated to CMV infection. In addition, hepatic fibrosis, ascites, growth retardation and impaired general condition were all evidenced. At 5 years old she was evaluated because epistaxis, multiple telangiectases on face, breathlessness, ascites, hypoxemia and evidence of multiple pulmonary arteriovenous fistulas. She was referred to our institution due to the case complexity. The patient was admitted to the pediatric intensive care unit. On physical examination, she presented a poor general status with growth retardation, heart rate of 80 beats per minute, respiratory rate of 25 breaths per minute, blood pressure 96/55 mmHg, temperature 36.2^o^ C, oxygen saturation 84%, weight 12.5 kg and height 88 cm. Multiple telangiectases on face, hypertelorism and low-set ears were evidenced. Supplementary oxygen was necessary with a 60% Venturi device. Cardiopulmonary examination showed a grade II/IV systolic heart murmur in mesocardium. Pulmonary auscultation was normal. In the abdominal examination an enlarged liver (3 cm below the right costal margin) without splenomegaly was documented. Limbs with drumstick-like fingers, capillary refill of two seconds, peripheral and central cyanosis were also observed. Neurological examination was normal.

**Figure 1:**
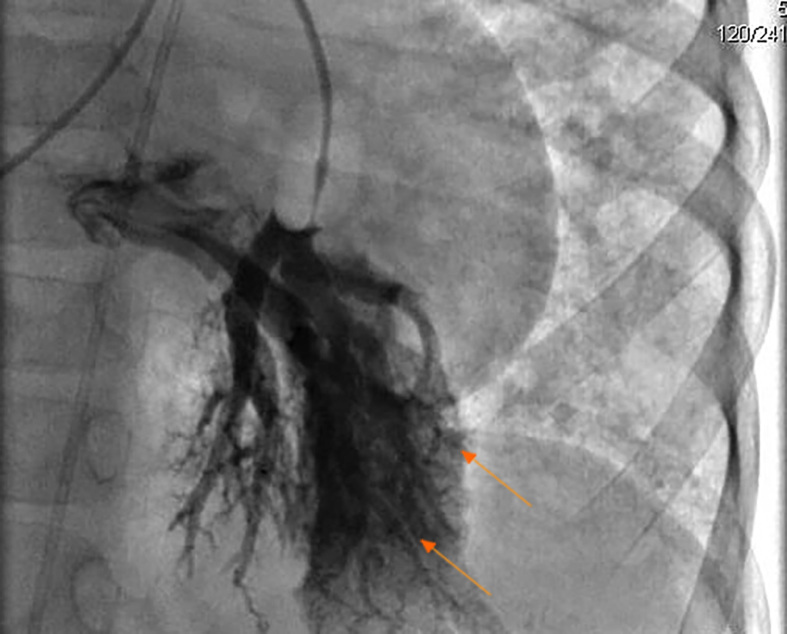
Cardiac catheterization showed arteriovenous malformations (arrows) on the left lung in the patient with Hereditary hemorrhagic telangiectasia.

Extensive studies were completed. At admission: white blood cell count 14,540/µL, neutrophils 8,840/µL, lymphocytes 4,420/µL, monocytes 1,060/µL, hemoglobin 15.2 g/dL, platelets 136,000/µL. Acute-phase reactants were normal (C-reactive protein of 0.37 mg/dL and erythrocyte sedimentation rate in 18 mm/h), Electrolytes were normal. Hepatic involvement was documented with a total bilirubin of 1.78 mg/dL, indirect 1.01 mg/dL and direct 0.77 mg/dL, in addition to abnormal Gamma glutamyl transferase (220 ​​U/L, normal value up to 41 U/mL). Transaminases were also altered (AST 71.2 U/L and ALT 53.6 U/L). Chemical analysis showed a normal renal function; total proteins of 5.90 g/dL, albumin 3.17 g/dL and Ferritin 291.7 ng/mL (reference value 4-67 ng/mL). Acute infectious diseases were all discarded (IgG isotype to CMV, Toxoplasma and Rubella were positive). Immunological analysis showed a positive response to 50% of serotypes 7-14 to *Streptococcus pneumoniae* for Prevenar 7. High levels of IgE were evidenced. On the other hand, IgA, IgG and IgM total levels were normal. Levels of C3 and C4 were normal. Phenotypic lymphocytic counts were normal (CD3+: 3542/ µL-77%- of lymphocytes, with a ratio CD4+/CD8+: 1). Antinuclear, anti-mitochondrial, and anti-smooth muscle antibodies were all negative. Enzymatic analysis of α-1-antitrypsin and α-fetoprotein were normal. Biopsy of the liver and spleen showed perisinusoidal and septal IV/VI fibrosis, extramedullary hematopoiesis, and portal hypertension. All microbiological analysis were negative. Bone marrow biopsy was normal. Several imaging studies were done, including a normal brain Magnetic Resonance Angiography. Thorax tomography showed abnormal dilation of pulmonary veins, and growth of right cavities. Splenoportal angioresonance of hepatic circulation showed nodular regenerative changes without evidence of focal lesions of neovasculature, and permeable splenic-portal circulation. Endoscopy showed hypertensive gastropathy and esophageal varices grade I-II. Echocardiogram showed multiple secondary intrapulmonary shunts with a positive bubble contrast test; systolic and diastolic functions preserved, mild pulmonary stenosis, peak gradient of 36 mmHg, pulmonary artery pressure in 44 mm Hg and left ventricular ejection fraction of 73%. Abdominal ultrasound showed hepatic nodular regenerative changes, and splenomegaly. Splenoportal Doppler ultrasound described no evidence of vascular abnormalities and portal hypertension with preserved flow. A diagnostic cardiac catheterization was done, showing multiple AVMs in all lung lobes, predominantly on the left lung (Fig. 1). 

During hospitalization, the patient presented several episodes of epistaxis, and deterioration of cardiopulmonary function with higher oxygen requirements secondary to pulmonary arteriovenous shunts. Gastrointestinal, liver or central nervous system shunts were discarded. Because of episodes of spontaneous and recurrent epistaxis, and the presence of multiple telangiectases and pulmonary AVMs, the diagnosis of HHT (Rendu-Osler-Weber) syndrome was made, despite of having no family history. Immunodeficiencies or lymphoproliferative disorders were excluded. Hepatopulmonary syndrome (HPS) was also ruled out based on the presence of pulmonary AVMs, multiple telangiectases, and epistaxis that were compatible with HHT over HPS. Patient required high-flow oxygen, bronchodilators and antihypertensive treatment. Five catheterizations for management were required with the implantation of more than 60 coils. The initial interventional catheterization treated AVMs localized on the right inferior lobe (15 embolizations). The second and the third catheterizations were done in the left inferior lobe and lingula. In the fourth catheterization, recurrence was evidenced in the left lung and new AVMs were found in the right inferior lobe requiring new embolizations. 

Due to a regular development and emergence of new arteriovenous communications during cardiac catheterization, in a 6-month period, a systemic treatment was planned. Among the alternatives proposed in the literature, the use of estrogen, thalidomide and bevacizumab were all considered. Because of the age of the patient, the potential toxicity and teratogenicity the use of the first two treatments was discarded. Due to the successful treatment in adult patients with HHT and, after multiple meetings conducting by all the medical specialties, ethics committee of the institution, parents and her health service authorization, we decided to start bevacizumab. Bevacizumab was administered at doses of 5 mg/kg (65 mg) every 15 days. After an 8-month hospitalization, and the infusion of 6 doses of bevacizumab, the patient improved the respiratory symptoms, epistaxis episodes and evolution of arteriovenous malformations without recurrence in the last cardiac catheterization. At discharge, the oxygen requirement decreased from 60% FiO2 to 28%, with an oxygen saturation of 80%. Facial telangiectases improved significantly. Echocardiogram showed also improvement with decreasing pulmonary artery pressure from 44 mmHg to 22 mm Hg and reduction in peak gradient of 36 mmHg to 17 mmHg (Table 1). Thoracic CT showed no new AVMs. 

The drug infusions were well tolerated, with no evidence of infection or adverse effects associated with the injection. After one year of follow-up, the clinical condition of the patient remains stable. 

**Table 1:** Echocardiography findings after cardiac catheterizations and embolotherapy.

Catheterization	Time in relation with bevacizumab	Aortic valve Peak gradient (mmHg)	Pulmonary valve peak gradient (mg Hg)	Tricuspid valve	Pulmonary artery pressure (mg Hg)
1	4 months before	32.0 (without insufficiency)	36	Mild to Moderate insufficiency	44
2	3 months before	33.0	32	Mild to moderate insufficiency	42
4	Bevacizumab treatment	28.5	23	Mild insufficiency	32
6	3 months after finish the treatment	27.0	17	Mild insufficiency	22

After the third and fifth cardiac catheterization, no echocardiography was done. Last echocardiography was done at discharge after 6 cycles of bevacizumab. 

## Discussion 

HHT is an autosomal dominant disorder, which includes a wide spectrum of mutations and genes involved. According to the genetic abnormality, different organs may be involved. In addition, there is a high incidence of *de novo* mutations ^3^. The HHT diagnosis is made by three of the four Curaçao criteria (epistaxis, multiple telangiectases, visceral gastrointestinal, lung, liver and/or brain lesions, and first-degree relative with HHT) ^18^. If three or four are met, a patient has "definite HHT". Our patient had no first-degree relative with HHT but the other three criteria were present. Other diagnosis such as HPS were ruled out based on the presence of pulmonary AVMs and multiple telangiectases and epistaxis that were compatible with HHT. Bevacizumab is an antiangiogenic drug, used mostly as an antineoplastic, decreasing the progression and formation of new vessels. The drug is approved by the FDA for the management of metastatic disease mainly in cervical cancer, glioblastoma, small cell lung and colorectal cancer ^15^, ^19^. Concerning the HHT, bevacizumab has been used in case reports and series, showing a good outcome with improvement of cardiac index in 87.5% of patients, and reducing dyspnea and episodes of epistaxis from 26 to 6 per month, due to the decrease of the AVMs. Secondary to cardiopulmonary function improvement, the need for liver transplantation in these patients has decreased; and the quality of life in the emotional, physical and vitality aspects improved greatly at 6 months after initiation of treatment ^17^, ^20^. Although the most important mechanism of action is the inhibitory effect over VEGF, thus inhibiting proliferation of endothelial cells, endothelial growth and cause regression of existing vessels increasing endothelial cell death; other mechanisms have been proposed, including prevention of VEGF-induced vessel permeability, lowering blood inflow through A-V shunts, decrease in cardiac output in patients with severe hepatic vascular malformations and improvement of anemia by reducing epistaxis and gastrointestinal bleeding ^21^ ^-^ ^23^. These indirect mechanisms may explain the improvement on facial telangiectases and the oxygen levels seen in our patient. The comparative benefits among bevacizumab *vs*. AVM occlusion concerning this case are also discussed in Table 2. 

**Table 2:** Discussion of benefits and limitations of embolotherapy in the management of arteriovenous malformations in hereditary hemorrhagic telangiectasia globally and in our patient. The left column lists the potential benefits and limitations of embolotherapy, in the column on the right, these parameters are discussed in relation to our case report. The evidence listed corresponds to other case reports.

Embolotherapy benefits in HHT-AVM	Results in the reported case
PAVM occlusion can eliminate or reduce PAVM-related shunt effect, improving blood oxygenation.	Although little clinical effect was evidenced after several cardiac catheterization and embolizations, emergence of new AVMs was evidenced. After bevacizumab treatment an important clinical improvement in blood oxygenation was seen. Unfortunately, we did not develop a new cardiac catheterization due to the stable clinical condition, thus we cannot demonstrate the PAVM involution. In angio-CT no new AVMs were found. Thus, a direct mechanism may be elucidated. In addition, indirect mechanisms of bevacizumab may be implicated in the clinical amelioration including decrease in cardiac output due to AVM control and improvement of anemia ^23^.
AVM occlusion can prevent possible future AVM-related brain abscess/stroke events.	To date, there is no evidence that bevacizumab will have better or equal performance concerning this issue. In our patient, no brain AVMs were evidenced and no cerebral accidents occurred 1 year after treatment. However a longer follow-up is required.
AVM occlusion may prevent future AVM enlargements.	No imaging studies are available after one year of follow-up in our patient. Therefore we do not have evidence that bevacizumab prevented possible enlargement of persistent AVMs. A longer follow-up is required.
Embolotherapy limitations in HHT-AVM	Results in the reported case
Complex AVMs, with multiple feeding arteries, may not be occluded completely.	In this case, it seems that bevacizumab gave better results than embolotherapy, as recanalization occurred after embolization. However, it is known that some recanalization events take place in complex PAVMs when too small feeding vessels cannot be effectively embolized, thus going towards enlargement some weeks/months after treatment. Usually, these collateral vessels are treated in a subsequent session with good outcome and often definite regression of the PAVM. It may be speculated that the fourth embolization session, permitted a definitive regression of the complex PAVMs, and that no further embolization would have been required to treat new PAVMs or re-perfused PAVMs. In this case, the observed amelioration would be due to a bevacizumab effect exerted on PAVM symptoms (possibly through alternative mechanisms) rather than on real PAVM involution.
Embolotherapy has a small, but significant risk of recurrence, especially in children.	Recurrence after embolotherapy has been reported up to 15% of cases ^24^. The fourth embolization was due to a reperfusion of an occluded vessel, supporting bevacizumab, as a mechanism to prevent recurrence risk in PAVM treatment. However, the follow-up is too short to draw a definitive conclusion.
PAVMs with diffuse pattern, according to Faughnan ^25^ and Pierucci ^26^, are refractory to embolotherapy and have no current therapeutic options, except lung transplantation.	Faughnan and Pierucci have shown that about 5% of PAVMs have a diffuse pattern (i.e., every subsegmental artery of a lobe has at least one PAVM). Embolization has shown to be quite ineffective in these cases. In our case no diffuse pattern was found. The use of bevacizumab in this subgroup of patients, who currently have no therapeutical option, deserves to be evaluated.

PAVM: pulmonary arteriovenous malformations; AVM: arteriovenous malformations; HHT: hereditary hemorrhagic telangiectasia.

This particular case was a big challenge for us because the local therapeutic alternatives were not enough to improve the clinical of the patient or the progress of the disease. The use of bevacizumab was proposed as a therapeutic off-label option where the local initiative therapies were insufficient. The good evolution of the patient after starting bevacizumab suggested its effectiveness as an antiangiogenic factor. 

The use of bevacizumab in the pediatric population has been limited to patients with cancer, for example neuroblastoma, Wilms' tumor, central nervous system tumors, vascular tumors ^27^. The most commonly reported adverse events in children have been lymphopenia , Rash, mucositis, proteinuria, arterial hypertension, defective wound healing , epistaxis, with an occurrence in 17% of patients ^28^. In the mentioned studies none pediatric patient needed to discontinue the drug due to the presence of adverse effects and its appearance was not associated with the duration of therapy ^29^ ^,^ ^30^. In our patient, these adverse effects were actively sought and none of them occurred during follow-up.

However, some considerations must be taken. Our patient seems to have a complex phenotype, HHT being part of a wider clinical spectrum. Hypertelorism, low-set ears, and growth retardation, as well mild pulmonary stenosis, might be due to a dysmorphologic syndrome. As we did not perform genetic analysis, a contiguous-gene deletion syndrome with a deletion encompassing ENG or ALK1/ACVRL1 (the well-known HHT causing syndrome) may be hypothesized. Hence, her failure to respond to embolotherapy might be due to the presence of a more complex syndrome than HHT alone. In this context, the benefic effects of bevacizumab may be related to the complex genetic condition, and no conclusion can be drawn to generalize bevacizumab effect in the whole HHT population. Genetic counselling was requested in our patient. 

Concerning mild pulmonary valve stenosis, an improvement was also observed. Thus another bevacizumab-related mechanism rather than AVMs improvement may affect the stenosis. This aspect needs to be further evaluated in other cases. 

In addition, our observational period (over 1 year) is too short to get definitive conclusions on bevacizumab effectiveness. Even other events, such as AVM-related brain abscesses that may be prevented with embolotherapy more than bevacizumab. We are following the clinical course of our patient. 

Through this case, a new systemic drug choice in pediatric patients with this syndrome is planted, showing an acceptable safety profile, decreasing progression of symptoms, and the need for liver transplantation and improve quality of life.
