# Supplementary material for: Bevacizumab as a treatment for hereditary hemorrhagic telangiectasia in children: a case report
Source: Colomb Med (Cali). 2017 Jun 30;48(2):88–93. (PMC5625558)
Supplement: Translation

# Translation: Bevacizumab como tratamiento para telangiectasia hemorrágica hereditaria en niños: Reporte de caso

## Introducción

La telangiectasia hemorrágica hereditaria (THH) o síndrome de Osler Weber Rendu es una enfermedad vascular multi sistémica autosómica dominante, caracterizada por telangiectasias mucocutaneas y malformaciones arteriovenosas (MAVs) que predisponen a formación de shunts y sangrados. La prevalencia es cercana a 1:5,000-8,000 ^1^. En la mayoría de casos es asociada a mutaciones en los genes que codifican para receptores de la superfamilia del factor de crecimiento transformante β/proteína morfogénica ósea (TGF-β/BMP). Las mutaciones identificadas causan disfunción endotelial, angiogenesis y/o remodelamiento vascular ^2^^,^^3^. Las mutaciones en dos genes diferentes son las responsables de los 2 subtipos: THH1 (mutación en gen ENG, principalmente MAVs con compromiso pulmonar y cerebral) y THH2 ( Mutación en Acvrl1, principalmente MAVs hepáticas) ^1^^-^^3^. 

El número y localización de las lesiones varían, incluso en la misma familia de los pacientes afectados. La mayoría de telangiectasias son en la mucosa oral, nasal y gastrointestinal ^4^; las MAVs pueden comprometer los pulmones ^5^, hígado ^6^, y sistema nervioso central ^7^. La epistaxis es generalmente, aunque no siempre, el signo más temprano ^1^^,^^2^. La manifestación inicial también puede ser absceso cerebral, hemorragia intracraneal o síntomas pulmonares ^8^. En pacientes pediátricos con THH, las manifestaciónes más comunes son epistaxis y compromiso mucocutaneo. Otros órganos afectados son pulmones y cerebro, con presencia de MAVs incluso en pacientes asintomáticos. MAVs grandes se asocian frecuentemente con complicaciones en la infancia ^8^^,^^9^. El compromiso hepático puede llevar a insuficiencia hepática y a falla cardiaca derecha, hipertensión portal y enfermedad biliar isquémica ^10^ Dado que actualmente no se dispone de una terapia sistémica eficaz, sólo se emplean tratamientos locales, según el órgano implicado. Por ejemplo, en el tratamiento de la epistaxis varias opciones como septodermoplastia, ligadura de arteria quirúrgica y láser, entre otros, son eficaces para el control local. Para el tratamiento de la hemorragia gastrointestinal, el uso de láser o sonda térmica han mostrado un beneficio a corto plazo ^4^^,^^11^. La embolización de MAVs del sistema nervioso central o pulmones ha demostrado ser una buena opción ^12^. Los pacientes asintomáticos con MAVs hepáticas no suelen ser tratados ^10^^,^^11^. Por otro lado, la terapia sistémica incluye estrógenos ^11^, tamoxifeno para el sangrado recurrente pero con restricciones debido a su efecto teratogénico ^13^; Y el ácido tranexámico que ha mostrado resultados prometedores pero con un potencial efecto protrombótico ^14^.

El bevacizumab, un anticuerpo monoclonal humanizado recombinante que inhibe el factor de crecimiento endotelial vascular (VEGF), se une a sus receptores en la superficie de las células endoteliales, inhibe la proliferación de células endoteliales, el crecimiento endotelial y causa la regresión de vasos existentes aumentando la muerte celular endotelial ^15^. El uso de bevacizumab intravenoso en dosis de 5-10 mg/kg cada tres semanas en THH ha demostrado disminución de episodios de epistaxis y mejoría del alto gasto cardíaco secundario a MAVs ^16^ y evita la necesidad de trasplante hepático después de seis meses de tratamiento en pacientes adultos con compromiso hepático en THH ^17^. En este artículo presentamos el caso de una paciente pediátrica con múltiples MAVs secundarias a THH, tratada con éxito con bevacizumab durante tres meses (6 ciclos). 

## Reporte de caso

Una paciente de 5 años de edad fue remitida a nuestra institución por insuficiencia hepática y múltiples fístulas arteriovenosas pulmonares. La madre fumó durante el primer trimestre del embarazo y se diagnosticó citomegalovirus congénito (CMV). El parto fue a las 38 semanas sin complicaciones, peso 2,500 g, longitud 42 cm. Dos meses después del nacimiento, el paciente desarrolló bronquiolitis obliterante y neumonía por CMV (carga viral >120,000 copias), deterioro de la función respiratoria y hemorragia alveolar. Requirió ventilación mecánica invasiva y terapia antiviral. En ese momento, presentó una función hepática anormal que se asoció a la infección por CMV. Además, se evidenció fibrosis hepática, ascitis, retraso del crecimiento y deterioro de la condición general. A los 5 años fue evaluada por epistaxis, telangiectasias múltiples en la cara, disnea, ascitis, hipoxemia y evidencia de múltiples fístulas arteriovenosas pulmonares. Ella fue remitida a nuestra institución debido a la complejidad del caso. La paciente fue ingresada en la unidad de cuidados intensivos pediátricos. En el examen físico, presentaba un pobre estado general con retraso del crecimiento, frecuencia cardiaca de 80 latidos por minuto, frecuencia respiratoria de 25 respiraciones por minuto, presión arterial 96/55 mmHg, temperatura 36.2^o^ C, saturación de oxígeno 84%, peso 12.5 kg y estatura de 88 cm. Se evidenciaron múltiples telangiectasias en la cara, hipertelorismo y orejas de implantación baja. Fue necesario tratamiento con oxígeno suplementario con un dispositivo Venturi a 60%. El examen cardiopulmonar evidenció un soplo cardíaco sistólico de grado II/IV en el mesocardio. La auscultación pulmonar fue normal. En el examen abdominal se documentó una hepatomegalia (3 cm por debajo del margen costal derecho) sin esplenomegalia. También se observó acropaquia, llenado capilar de 2 segundos, cianosis periférica y central. El examen neurológico fue normal.

Se completaron estudios de extensión. A la admisión: Leucocitos 14,540/μL, neutrófilos 8,840/μL, linfocitos 4,420/μL, monocitos 1,060/μL, hemoglobina 15.2/μL, plaquetas 136,000/μL. Los reactantes de fase aguda fueron normales (proteína C-reactiva de 0.37 mg/dL y velocidad de eritrosedimentacion en 18 mm/h), los electrolitos fueron normales. Se documentó compromiso hepático con una bilirrubina total de 1.78 mg/dL, bilirrubina indirecta 1.01 mg/dL y bilirrubina directa de 0.77 mg/dL, además de una gamma glutamil transferasa (GGT) anormal (220 U/L, valor normal hasta 41 U/L). Las transaminasas también estaban elevadas (AST 71.2 U/L y ALT 53.6 U/L). Los demás paraclínicos mostraron una función renal normal; Proteínas totales de 5.90 g/dL, albúmina 3.17 g/dL y ferritina 291.7 ng/mL (valor de referencia 4-67 ng/mL). Se descartaron enfermedades infecciosas agudas (los isotipos IgG para CMV, *Toxoplasma* y Rubéola fueron positivos). El análisis inmunológico mostró una respuesta positiva al 50% para los serotipos 7-14 a *Streptococcus pneumonie* para Prevenar 7. Se evidenciaron altos niveles de IgE. Por otro lado, los niveles totales de IgA, IgG e IgM fueron normales. Los niveles de C3 y C4 fueron normales. Los recuentos linfocíticos fenotípicos fueron normales (CD3+: 3,542/μL-77% - de linfocitos, con una relación CD4+/CD8+: 1). Los anticuerpos antinucleares(ANAS), anti-mitocondriales y anti-músculo liso fueron todos negativos. El análisis enzimático de α1-antitripsina y α-fetoproteína fue normal. La biopsia del hígado y el bazo mostraron fibrosis perisinusoidal y septal IV/VI, hematopoyesis extramedular e hipertensión portal. Todos los análisis microbiológicos fueron negativos. La biopsia de médula ósea fue normal. Se realizaron varios estudios de imagen, incluyendo una Angiografía por Resonancia Magnética cerebral normal. La tomografía de tórax mostró dilatación anormal de las venas pulmonares y crecimiento de cavidades cardiacas derechas. La angiorresonancia esplenoportal de la circulación hepática mostró cambios regenerativos nodulares sin evidencia de lesiones focales de neovasculatura y circulación esplénica-portal permeable. La endoscopia de vías digestivas altas mostró gastropatía hipertensiva y varices esofágicas grado I-II. El ecocardiograma mostró múltiples derivaciones intrapulmonares secundarias con una prueba positiva de burbujas con contraste, funciones sistólica y diastólica preservada, estenosis pulmonar leve, gradiente pico de 36 mmHg, presión arterial pulmonar en 44 mmHg y fracción de eyección del ventrículo izquierdo del 73%. La ecografía abdominal mostró cambios regenerativos nodulares hepáticos y esplenomegalia. La ecografía Doppler esplenoportal no describió evidencia de anomalías vasculares e hipertensión portal con flujo preservado. Se realizó un cateterismo cardíaco de diagnóstico, mostrando MAVs múltiples en todos los lóbulos pulmonares, predominantemente en el pulmón izquierdo (Fig. 1).

**Figura 1:** El cateterismo cardíaco mostró malformaciones arteriovenosas (flechas) en el pulmón izquierdo del paciente con telangiectasia hemorrágica hereditaria.

Durante la hospitalización, el paciente presentó varios episodios de epistaxis y deterioro de la función cardiopulmonar con mayores requerimientos de oxígeno secundarios a las derivaciones arteriovenosas pulmonares. Se descartó la presencia de derivaciones gastrointestinales, del hígado o del sistema nervioso central. Debido a los episodios de epistaxis espontánea y recurrente, y a la presencia de múltiples telangiectasias y MAVs pulmonares, se realizó el diagnóstico de síndrome HHT (Rendu-Osler-Weber), a pesar de no tener antecedentes familiares. Se excluyeron inmunodeficiencias o trastornos linfoproliferativos. El síndrome hepatopulmonar (HPS) también se descartó en base a la presencia de MAVs pulmonares, telangiectasias múltiples y epistaxis que eran compatibles con HHT sobre HPS. El paciente requirió oxígeno de alto flujo, broncodilatadores y tratamiento antihipertensivo. Se requirieron cinco cateterismos para el manejo, con la implantación de más de 60 coils. La intervención inicial con cateterismo trató las MAVs localizadas en el lóbulo inferior derecho (15 embolizaciones). El segundo y el tercer cateterismo se realizaron en el lóbulo inferior izquierdo y lingula. En el cuarto cateterismo, la recurrencia se evidenció en el pulmón izquierdo y se encontraron nuevas MAVs en el lóbulo inferior derecho que requirieron nuevas embolizaciones.

Debido a un desarrollo regular de nuevas comunicaciones arteriovenosas durante la terapia de cateterismo cardíaco en un período de seis meses, se planificó un tratamiento sistémico. Entre las alternativas propuestas en la literatura, se consideraron el uso de estrógenos, talidomida y bevacizumab. Debido a la edad del paciente, la toxicidad potencial y la teratogenicidad del uso de los dos primeros tratamientos estos se descartaron. Debido al éxito del tratamiento en pacientes adultos con THH y, después de múltiples reuniones realizadas por todas las especialidades médicas tratantes, el comité de ética de la institución, los padres y su autorización de servicio de salud, decidimos iniciar el bevacizumab. Bevacizumab se administró a dosis de 5 mg/kg (65 mg) cada 15 días. Tras una hospitalización de ocho meses y la infusión de 6 dosis de bevacizumab, la paciente presentó una mejoría en los síntomas respiratorios, episodios de epistaxis y en la evolución de malformaciones arteriovenosas sin recurrencia en el último cateterismo cardíaco. Al momento del alta, el requerimiento de oxígeno disminuyó del 60% FiO2 al 28%, con una saturación de oxígeno del 80%. Las telangiectasias faciales mejoraron significativamente. El ecocardiograma mostró también mejoría con disminución de la presión arterial pulmonar de 44 mmHg a 22 mm Hg y reducción en el gradiente pico de 36 mmHg a 17 mmHg (Tabla 1). La TAC torácica no mostró nuevas MAVs.

**Tabla 1:** Hallazgos de ecocardiografia despues de los cateterismos cardiacos y emboloterapia.

Cateterismo	Tiempo en relación al bevacizumab	Gradiente pico de válvula aortica mmHg	Gradiente pico de válvula pulmonar (mg Hg)	Válvula tricuspide	Presion de arteria pulmonar (mg Hg)
1	4 meses antes	32.0 (sin insuficiencia)	36	Insuficiencia leve a moderada	44
2	3 meses antes	33.0	32	Insuficiencia leve a moderada	42
4	Tratamiento con Bevacizumab	28.5	23	Insuficiencia leve	32
6	3 meses después de finalizar el tratamiento	27.0	17	Insuficiencia leve	22

Las infusiones del medicamento fueron bien toleradas, sin evidencia de infección o efectos adversos asociados con la inyección. Después de un año de seguimiento, el estado clínico del paciente permanece estable.

Después del tercer y quinto cateterismo cardiaco, no se realizó ecocardiografía. La última ecocardiografía se realizó al alta tras 6 ciclos de bevacizumab.

## Discusión

La THH es un trastorno autosómico dominante, que incluye un amplio espectro de mutaciones y genes implicados. Según la anomalía genética, pueden estar involucrados diferentes órganos. Además, existe una alta incidencia de mutaciones de novo ^3^. El diagnóstico de THH se realiza mediante tres de los cuatro criterios de Curaçao (epistaxis, telangiectasias múltiples, lesiones viscerales gastrointestinales, pulmonares, hepáticas y/o cerebrales y familiares de primer grado con THH) ^18^. Si se cumplen tres o cuatro pacientes, el paciente tiene una "THH definitiva": nuestro paciente no tenía pariente de primer grado con THH, pero los otros tres criterios estaban presentes. Otros diagnósticos como HPS fueron descartados en base a la presencia de MAVs pulmonares y múltiples telangiectasias y epistaxis que son compatibles con THH. El bevacizumab es un fármaco antiangiogénico, usado principalmente como antineoplásico, que disminuye la progresión y la formación de nuevos vasos. El fármaco es aprobado por la FDA para el manejo de la enfermedad metastásica principalmente en cáncer cervical, glioblastoma, cáncer de pulmón de células pequeñas y el cáncer colorrectal ^15^^,^^19^. En cuanto a la THH, el bevacizumab se ha utilizado en informes de casos y series, mostrando un buen resultado con la mejora del índice cardíaco en el 87.5% de los pacientes y la reducción de la disnea y episodios de epistaxis de 26 a 6 por mes, debido a la disminución de las MAVs. Secundario a la mejora de la función cardiopulmonar, la necesidad de trasplante hepático en estos pacientes ha disminuido, y la calidad de vida en los aspectos emocional, físico y de vitalidad mejoró mucho a los 6 meses después del inicio del tratamiento ^17^^,^^20^. Aunque el mecanismo de acción más importante es el efecto inhibidor sobre VEGF, inhibiendo así la proliferación de células endoteliales, el crecimiento endotelial y causando la regresión de vasos existentes que aumentan la muerte celular endotelial; Se han propuesto otros mecanismos, entre ellos la prevención de la permeabilidad vascular inducida por VEGF, la disminución de la afluencia de sangre a través de las derivaciones A-V, la disminución del gasto cardiaco en pacientes con malformaciones vasculares hepáticas severas y la mejora de la anemia mediante la reducción de la epistaxis y el sangrado gastrointestinal ^21^^,^^23^. Estos mecanismos indirectos pueden explicar la mejora de las telangiectasias faciales y los niveles de oxígeno observados en nuestro paciente. Los beneficios comparativos entre el bevacizumab frente a la oclusión AVM en este caso también se discuten en la Tabla 2.

**Tabla 2:** Comparación entre emboloterapia y THH. Discusión de los beneficios y limitaciones de la emboloterapia en el manejo de malformaciones arteriovenosas en la telangiectasia hemorrágica hereditaria a nivel mundial y en nuestro paciente. En la columna de la izquierda se enumeran los posibles beneficios y limitaciones de la emboloterapia, en la columna de la derecha, estos parámetros se discuten en relación con nuestro caso. La evidencia que se muestra corresponde a otros informes de casos.

Beneficios de emboloterapia en THH y MAVs	Resultados en el reporte de caso
La oclusión de MAVPs puede eliminar o reducir el efecto de derivación relacionado con PAVM, mejorando la oxigenación de la sangre.	Aunque se evidenció poco efecto clínico después de varios cateterismos cardíacos y embolizaciones, se evidenció la aparición de nuevas MAVs. Después del tratamiento con bevacizumab se observó una importante mejoría clínica en la oxigenación sanguínea. Desafortunadamente, no desarrollamos un nuevo cateterismo cardíaco debido a la condición clínica estable, por lo que no podemos demostrar la involución de MAVPs. En el angio TAC no se encontraron nuevas MAVs. Por lo tanto, un mecanismo directo del bevacizumab puede ser la causa. Además, los mecanismos indirectos de bevacizumab pueden estar implicados en el mejoramiento clínico incluyendo la disminución del gasto cardíaco debido al control de MAVs y la mejoría de la anemia ^23^.
La oclusión de MAVs puede prevenir los abscesos y ACV relacionados a ellas.	A la fecha, no hay evidencia de que el bevacizumab tenga mejor o igual rendimiento en relación a esto. En nuestro paciente, no MAVs cerebrales fueron evidenciadas y no se produjeron accidentes cerebrales 1 año después del tratamiento. Sin embargo, se requiere un seguimiento más largo.
La oclusión de las MAVs puede prevenir su futuro crecimiento.	No hay estudios de imagen disponibles después de un año de seguimiento en nuestra paciente. Por lo tanto, no disponemos de pruebas de que el bevacizumab prevenga el posible crecimiento de las MAVs persistentes. Se requiere un seguimiento más largo.
Limitaciones en emboloterapia HHT-AVM	Resultados en el reporte de caso
Las MAVs complejas, con múltiples arterias nutricias, pueden no ser completamente ocluidas.	En este caso, parece que el bevacizumab dio mejores resultados que la emboloterapia, la recanalización se produjo después de la embolización. Sin embargo, se sabe que algunos eventos de recanalización tienen lugar en MAVPs complejas cuando no se pueden embolizar eficazmente los vasos nutricios demasiado pequeños, lo que hace que haya un aumento de tamaño algunas semanas/meses después del tratamiento. Por lo general, estos vasos colaterales se tratan en una sesión posterior con buen resultado y a menudo una regresión definitiva de las MAVPs. Se puede especular que la cuarta sesión de embolización permitió una regresión definitiva de las MAVPs complejas y que no se requerirá ninguna embolización adicional para tratar nuevas MAVPs o MAVPs re-perfundidas. En este caso, la mejoría observada se debe a un efecto de bevacizumab ejercido sobre las MAVPs (posiblemente a través de mecanismos alternativos) en lugar de sobre la involución real de las MAVPs.
La emboloterapia tiene un pequeño pero significativo riesgo de recurrencia, especialmente en niños.	La recurrencia después de la emboloterapia ha sido reportada hasta el 15% de los casos ^(^^24^. La cuarta embolización se debió a una reperfusión de un vaso ocluido, apoyando el bevacizumab, como un mecanismo para prevenir el riesgo de recurrencia en el tratamiento con MAVPs. Sin embargo, el seguimiento es demasiado corto para llegar a una conclusión definitiva.
MAVPs con patrón difuso, de acuerdo con Faughnan ^25^ y Pierucci ^26^, son refractarios a la embolización y actualmente no tienen opciones terapéuticas actuales, a excepción de trasplante de pulmón.	Faughnan y Pierucci han mostrado que aproximadamente el 5% de las MAVPs tienen un patrón difuso (por ejemplo, cada arteria subsegmentaria de un lóbulo tiene al menos una MAVPs). La embolización ha demostrado ser bastante inefectiva en estos casos. En nuestro caso no se encontró patrón difuso. El uso de bevacizumab en este subgrupo de pacientes, que actualmente no tienen opción terapéutica, merece ser evaluado.

MAVPs: Malformaciones arteriovenosas pulmonares AVM: Malformaciones arteriovenosas THH: Telangiectasia hemorrágica hereditaria

Este caso particular fue un gran desafío para nosotros porque las alternativas terapéuticas locales no fueron suficientes para mejorar la clínica del paciente o el progreso de la enfermedad. El uso de bevacizumab se propuso como una opción terapéutica off-label donde las terapias locales eran insuficientes. La buena evolución del paciente después del inicio del bevacizumab sugirió su eficacia como factor antiangiogénico.

El uso de bevacizumab en la población pediátrica se ha limitado a pacientes con cáncer, por ejemplo, neuroblastoma, tumor de Wilms, tumores del sistema nervioso central, tumores vasculares ^27^. Los eventos adversos más frecuentes en niños han sido linfopenia, erupción cutánea, Mucositis, proteinuria, hipertensión arterial, cicatrización de heridas defectuosa, epistaxis, con una incidencia en el 17% de los pacientes ^28^. En los estudios mencionados, ningún paciente pediátrico necesitó interrumpir el fármaco debido a la presencia de efectos adversos y su aparición no estuvo asociada con la duración del tratamiento ^29^^,^^30^. En nuestro paciente, estos efectos adversos fueron buscados activamente y ninguno de ellos ocurrió durante el seguimiento.

Sin embargo, algunas consideraciones deben tomarse. Nuestro paciente parece tener un fenotipo complejo, con la THH siendo parte de un espectro clínico más amplio. El hipertelorismo, las orejas de implantación baja y el retardo del crecimiento, así como la estenosis pulmonar leve, pueden deberse a un síndrome dismorfológico. Como no se realizó el análisis genético, un síndrome de deleción de genes contiguos con una deleción que abarque ENG o ALK1/ACVRL1 (el bien conocido como causante de THH) puede ser la hipótesis. Por lo tanto, su falta de respuesta a la emboloterapia podría deberse a la presencia de un síndrome más complejo que THH solo. En este contexto, los efectos benéficos del bevacizumab pueden estar relacionados con la compleja condición genética, y no se puede sacar ninguna conclusión para generalizar el efecto del bevacizumab en toda la población THH. El asesoramiento genético fue solicitado en nuestro paciente.

En cuanto a la estenosis de válvula pulmonar leve, también se observó una mejoría. Por lo tanto, otro mecanismo relacionado con el bevacizumab además de la mejora de la MAVs puede incidir sobre la estenosis. Este aspecto debe ser evaluado más a fondo en otros casos.

Además, nuestro período de observación (alrededor de 1 año) es demasiado corto para obtener conclusiones definitivas sobre la efectividad del bevacizumab. Incluso otros eventos, como MAVs relacionadas con abscesos cerebrales que se pueden prevenir con emboloterapia más que con bevacizumab. Estamos siguiendo el curso clínico de nuestro paciente.

A través de este caso, se plantea una nueva elección sistémica de fármacos en pacientes pediátricos con este síndrome, mostrando un perfil de seguridad aceptable, disminuyendo la progresión de los síntomas, la necesidad de trasplante hepático y mejorando la calidad de vida.
